# Long-Term Reduction of Short-Wavelength Light Affects Sustained Attention and Visuospatial Working Memory With No Evidence for a Change in Circadian Rhythmicity

**DOI:** 10.3389/fnins.2020.00654

**Published:** 2020-07-03

**Authors:** Aleksandra Domagalik, Halszka Oginska, Ewa Beldzik, Magdalena Fafrowicz, Malgorzata Pokrywka, Piotr Chaniecki, Marek Rekas, Tadeusz Marek

**Affiliations:** ^1^Brain Imaging Core Facility, Malopolska Centre of Biotechnology, Jagiellonian University, Kraków, Poland; ^2^Department of Cognitive Neuroscience and Neuroergonomics, Institute of Applied Psychology, Jagiellonian University, Kraków, Poland; ^3^Department of Clinical Biochemistry, Jagiellonian University Medical College, Kraków, Poland; ^4^Ophthalmology Private Practice, Kraków, Poland; ^5^Ophthalmology Department, Military Institute of Medicine, Warsaw, Poland

**Keywords:** short wavelength light, blue light, visuospatial memory, sustained attention, circadian rhythmicity

## Abstract

The short wavelength, i.e., blue light, is crucial for non-image forming effects such as entrainment of the circadian system in humans. Moreover, many studies showed that blue light enhances alertness and performance in cognitive tasks. However, most scientific reports in this topic are based on experiments using short exposure to blue or blue-enriched light, and only a few focused on the effects of its reduced transmittance, especially in longer periods. The latter could potentially give insight into understanding if age-related sleep problems and cognitive decline are related to less amount of blue light reaching the retina, as the eyes’ lenses yellow with age. In this study, we investigated the effects of prolonged blocking of blue light on cognitive functioning, in particular—sustained attention and visuospatial working memory, as well as on sleep, and melatonin and cortisol levels. A group of young, healthy participants was randomly allocated to either blue light blocking or control group. Depending on the group, participants wore amber contact lenses, reducing the transmittance of blue light by ∼90% or regular contact lenses for a period of 4 weeks. No changes were observed for measurements related to sleep and sleep–wake rhythm. Dim light melatonin onset, evening levels of melatonin, and morning cortisol answer did not show any significant alterations during blue light (BL) blockade. The significant effects were revealed both for sustained attention and visuospatial memory, i.e., the longer blocking the blue light lasted, the greater decrease in performance observed. Additionally, the follow-up session conducted ∼1 week after taking off the blue-blocking lenses revealed that in case of sustained attention, this detrimental effect of blocking BL is fully reversible. Our findings provide evidence that prolonged reduction of BL exposure directly affects human cognitive functioning regardless of circadian rhythmicity.

## Introduction

Humans adapted their life to 24 h light–dark cycle. As a diurnal species, we are exposed to light that is necessary not only for vision but also constitutes a powerful modulator of non-visual functions. The non-visual (or non-image forming, NIF) effects of light such as circadian rhythms regulation and pupil constriction are mediated by a retinal photoreceptor system built of the intrinsically photosensitive retinal ganglion cells (ipRGC; Zaidi et al., 2007; Güler et al., 2008) probably due to direct neuronal projections of these cells to many hypothalamic nuclei, including suprachiasmatic nuclei (master circadian pacemaker) and olivary pretectal nuclei (Hattar et al., 2006). Human and animal studies provide evidence that the NIF system detects variations in ambient irradiance and elicits long-term modifications of circadian rhythms as well as acute changes in hormone secretion, heart rate, sleep propensity, alertness, core body temperature, retinal neurophysiology, pupillary constriction, and gene expression (reviewed by Vandewalle et al., 2009; Duda et al., 2020).

In these unique ipRGC cells, the triggering signal transduction is accomplished by the photopigment melanopsin, which shows maximum sensitivity to the blue, i.e., short wavelength, part of the spectrum (∼480 nm; Berson et al., 2002; Hattar et al., 2002). Behavioral, biochemical, and neuroimaging studies, as well as subjective measurements, demonstrated that the sensitivity of the human circadian system and alerting and cognitive responses to light is blue-shifted relative to the three-cone visual photopic system, thus related to melanopsin phototransduction (see reviews by Cajochen, 2007; Vandewalle et al., 2009; Chellappa et al., 2011a).

Here, we focus on alerting and cognitive functions, and how these are affected by changed light condition. Studies demonstrated that exposure to monochromatic blue light improves performance, i.e., participants respond faster and with better accuracy, and at the same time reduces sleepiness in terms of subjective ratings (Lockley et al., 2006) or stimulates higher cognitive brain activity, independently of vision (Vandewalle et al., 2013). Recently, Alkozei et al. (2016) have shown that exposure to blue vs. amber (placebo) light led to better performance on a working memory task and increased functional brain responses related to the memory process. Later, the same group used verbal learning test and demonstrated better subsequent memory recall in the participants exposed to blue light during memory consolidation when compared to individuals exposed to an amber light condition (Alkozei et al., 2017). Recently, another neuroimaging study revealed that melanopsin-based photoreception activates a cerebral network including frontal regions, classically involved in attention and oculomotor responses (Hung et al., 2017). A range of studies introduced blue-enriched white light and showed that it improves subjective alertness and performance (Viola et al., 2008), speeds response times (Newman et al., 2016), and is more effective in reducing subjective sleepiness and enhancing cognitive performance, specifically associated with tasks of sustained attention (Chellappa et al., 2011b).

Most studies focusing on the effects of short-wavelength light present the experiments with exposure to blue or blue-enriched light. In addition, in most cases, they report the effect of short-lasting exposure to light (from short pulses to minutes and to hours). However, taking into account the aging process of the eye lens, it is also important to consider the condition of filtering the blue light for a prolonged time (days or weeks). During aging, the natural lens becomes more yellow because of the accumulation of chromophores that decrease transmission especially in the short-wavelength range of the visible spectrum (Giménez et al., 2010; Kessel et al., 2010). Interestingly, the transmission of light at 480 nm decreases by 72% from the age of 10–80 years (Kessel et al., 2010). A large sample study shows that while the age-related lens yellowing is of relatively little importance for visual function, it may be responsible for sleep problems in the elderly because of disturbed photoentrainment of circadian rhythms (Kessel et al., 2011). The authors reported the inverse relationship between blue light lens transmission and the risk of having sleep disturbances and concluded that filtration of blue light is the cause of disturbance of photoentrainment of circadian rhythms and sleep disturbances that are often observed in the elderly population (Foley et al., 2004). It was also shown that in this population, there is reduced responsiveness to short-wavelength light in terms of subjective alertness and sleepiness (Sletten et al., 2009). A later study on the consequences of cataractous lens replacement revealed that an increase in light input through the eye normalizes the advanced phases of sleep and melatonin that are often observed in elderly (Giménez et al., 2016). Additionally, a functional magnetic resonance study showed that the effect of blue light on brain responses diminishes with aging in areas typically involved in visual functions and in key regions for alertness regulation and higher executive processes (Daneault et al., 2014).

Therefore, studying the effects of reduced exposure to blue light is an important aspect of understanding the changes related to aging process. Only a few studies reported the consequences of short-term BL filtering using the short-wavelength attenuated polychromatic white- or blue-light blocking glasses. It was shown that this reduction leads to decrement of performance, subjective vigilance and efficiency, and affects physiological parameters linked to sleepiness and vigilance (van de Werken et al., 2013) as well as attenuation of LED-induced melatonin suppression in the evening and decreased vigilant attention and subjective alertness before bedtime (van der Lely et al., 2015). Blue-blocking glasses used in particular times of the day proved to change circadian measures such as sleep onset (shown in the study on delayed sleep phase disorder patients, Esaki et al., 2016) or sleep time and efficiency (see shift-work studies by Sasseville et al., 2009; Sasseville and Hébert, 2010). A prolonged reduction in short wavelength was introduced by Giménez et al. (2014) in the study on melatonin and sleep patterns. They used soft orange contact lenses (reducing ∼50% in short-wavelength range) for 2 weeks in order to mimic, to a certain extent, the aging effects of the lens’ yellowing in healthy young participants. After this period, the melatonin measures did not change, and the effects on sleep parameters were limited.

The goal of the current study was to observe the long-term effects of blue light filtering. In contrast to the aforementioned study by Giménez et al. (2014), we used lenses that reduced transmittance of blue light by ∼90% and introduced them for 4 weeks. We focused on the alertness (as one aspect of sustained attention) defined as the ability to achieve and maintain a certain level of cognitive performance in a given task as well as on visuospatial working memory performance. Participants were tested once a week with Psychomotor Vigilance task and sequential picture location task. Actigraphy measurements as well as Pittsburgh Sleep Quality Index and Epworth Sleepiness Scale were used to control for factors related to sleep and sleep–wake rhythm. Additionally, morning levels of saliva cortisol and evening levels of saliva melatonin were assayed. We hypothesized that our experimental condition causes a progressive deterioration of performance similar to the effect of aging-related cognitive decline, which might be, to some extent, linked to a reduced amount of blue light reaching the retina. Furthermore, to investigate whether these changes are reversible, we introduced a follow-up session 1 week after returning to normal light conditions.

The study was registered in the International Standard Randomized Controlled Trial Number (ISRCTN) clinical trial registry (number ISRCTN18109340; Marek, 2019).

## Materials and Methods

### Participants

**FIGURE 1:**
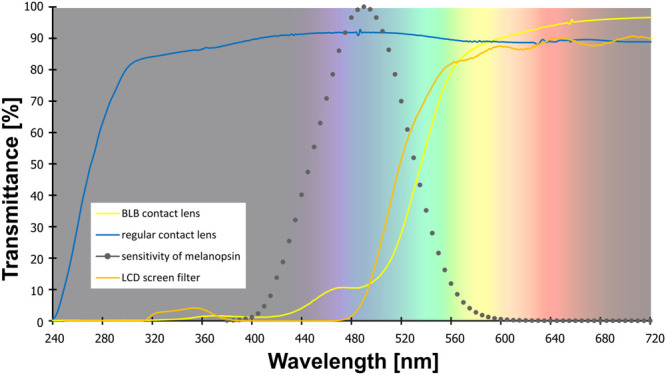
The transmittance of the contact lenses and filter used in the study. Note: melanopsin sensitivity adapted from Irradiance Toolbox (Lucas et al., 2014).

Forty-eight healthy participants [average age, 24.3 years (SD, 3.8); 32 female] started the experiment and were divided into two groups differing in the type of contact lenses used: blue light blocked (BLB) group and control (CTRL) group (parallel group study with balanced randomization 1:1). Participants were not aware of which group they would be assigned to; allocation was performed by a member of the research team by lot separately for each 6-week round of experiment (maximum number of participants for each round was 10, i.e., 5 for each group). All of them had nearsightedness (myopia) and good experience with contact lenses wearing. Each participant underwent a thorough ophthalmologic examination to exclude other sight problems and passed the Ishihara test for color blindness. Participants assigned to BLB group wore the amber contact lenses reducing the transmittance of BL by ∼90% on the 24 h basis (UltraVision, Igel RX; water content, 77%; orange tint density, 40%), whereas CTRLs wore the regular contact lenses (see filter properties in Figure 1). They were allowed to remove lenses for cleaning once a week if needed. The lenses were adjusted to participants’ refractive error. All participants were under ophthalmologist care throughout the whole experiment. All participants were right-handed, with no neurological or psychiatric disorders and drug-free. During selection procedure, we excluded individuals with poor sleep quality (Pittsburgh Sleep Quality Index, PSQI > 6; Buysse et al., 1989) and elevated level of daytime sleepiness (Epworth Sleepiness Scale scores, ESS > 10; Johns, 1991). The chronotype was assessed with the Chronotype Questionnaire (Oginska et al., 2017); extreme chronotypes were excluded. See Supplementary Figure S1 for details of enrollment. None of the participants worked night shifts or traveled across more than two time zones in the previous 2 months. Participants were financially rewarded for their participation, were informed about the procedure, and gave their written consent. The study was approved by the bioethics commission at the Polish Military Institute of Aviation Medicine and conducted in accordance with ethical standards described in the Declaration of Helsinki.

To avoid any expectancy effect, a possible positive or negative influence of the blue light filtering on functioning and the general well-being was not suggested to participants before the start of the experiment. They were informed that the *research goal* was primarily the observation of cognitive functioning in the situation of “sharpening the eye.” After the experiment, its true purpose was explained to the participants.

There were eight dropouts caused by discomfort and/or eye irritation (seven of them initially assigned to BLB group). Two subjects were excluded because of an elevated level of daytime sleepiness throughout the whole experiment and particularly high ESS score in baseline session; they were from the CTRL group. Due to dropouts, exclusion of participants, or technical problems, data from different number of participants were analyzed in used measures (exact numbers for each analysis are stated in section “Results”).

### Experimental Protocol

**FIGURE 2:**
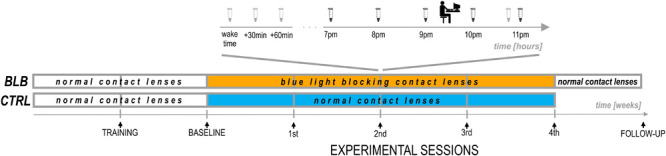
Experimental protocol.

The experiment lasted 6 weeks (Figure 2). For each participant, the measurements were obtained once a week on the same day of the week in the evenings. Participants from the CTRL group performed the task approximately at 7:30 P.M. and those from the BLB group approximately at 9:30 P.M. The timing of the test differed between groups, however, it was the same in every session for each group. The goal was to examine two people from both groups on the same day in order to make sure they were exposed to the same photoperiod. To check whether this discrepancy did not introduce bias regarding circadian and/or homeostatic factors, we have compared the results from the baseline session between groups. No significant differences were observed; thus, the time difference between acquisition did not affect our results.

For the first 2 weeks, all participants wore regular, daily disposable contact lenses. After the first week, participants familiarized themselves with a complete experimental protocol including experimental tasks (training session). The session after the second week was treated as a baseline. For the next 4 weeks, participants wore monthly disposable contact lenses with proper filter properties according to the group. They visited the laboratory after each week to complete experimental sessions. Additionally, participants from the BLB group had one more session (follow-up) ∼1 week after the main experiment; testing was performed in the evening hours (around 5–9 P.M.).

The participants were told to follow their preferred sleep–wake habits and work schedules, but to refrain from intensive/strenuous physical activity (running, cycling, gym) directly before the session. They were also asked to abstain from alcohol and caffeine during 24 h preceding the measurements. Chocolate, bananas, and citrus fruits or juices were not allowed on the day of examination. The experiment was conducted during spring to fall months ensuring greater availability of sunlight.

At each session, participants performed two experimental tasks in front of the computer screen and filled in the ESS questionnaire. They were also asked about visual sensations and discomfort in the preceding week; one of the goals of this interview was to monitor compliance of lens wear. During baseline and on the last experimental session, participants additionally fulfilled the PSQI questionnaire. Through the whole experiment (6 weeks), they wore actigraphs (AMI – Ambulatory Monitoring Inc. or MotionWatch8 – CamNtech Ltd.) on their non-dominant wrist. From the recordings, the following parameters were calculated: sleep onset and offset, sleep onset latency (calculated as the difference between sleep onset and “light-off” time marked with key-press by the participant), and actual sleep time. Additionally, for 19 participants that wore MotionWatch8 actigraphs (11 from BLP and 8 from CTRL group), the non-parametric circadian analysis was performed (Van Someren, 1997; Gonçalves et al., 2014). Three parameters were derived from this approach: intradaily stability, an indicator of the stability of the rest–activity cycle; interdaily variability, a marker of fragmentation of activity; and relative amplitude, a measure of the amplitude of the rest–activity cycle. No sleep diaries were used. To estimate daily exposure to sunlight, participants were asked how long and at what times they stayed outdoors.

At each session, participants from the BLB group provided saliva samples using a passive drool method (Salimetrics, LLC.; see Figure 2). For cortisol assay, they collected three samples in the morning (first one just after awakening and before eating breakfast and brushing teeth, the second 30 min after awakening, and the third 60 min after awakening) and one sample in the evening, around 10:50 PM. Participants were asked to keep morning samples refrigerated and to bring them to the lab in the evening. For melatonin assay, five samples were collected – every hour from 7 to 11 P.M. For this time, participants were asked to sit quietly, under dim lighting conditions (<5 lx). They rinsed their mouth with water 10 min before each sample and were not allowed to eat or drink 30 min before sampling. The samples were frozen and stored below −18°C. Salivary melatonin and cortisol were measured with competitive ELISA (salivary melatonin enzyme immunoassay kit and salivary cortisol enzyme immunoassay kit, Salimetrics^®^, Carlsbad, United States). Before analysis, all samples were vortexed and centrifuged at 1,500 × *g* for 15 min to get rid of mucins, which may affect antigen binding to the antibody and lead to incorrect results. The analysis was carried out according to the manufacturer’s protocol. Into appropriate wells of a microtiter plate, coated with antimelatonin or anticortisol monoclonal antibodies, standards, controls, and saliva samples were pipetted. Then, into each well, enzyme conjugate, containing melatonin or cortisol conjugated to horseradish peroxidase enzyme, was added, and plates were incubated for 3 h at 6°C in case of melatonin assay or for 1 h at room temperature in case of cortisol assay. After washing, into each well, tetramethylbenzidine substrate solution was pipetted, and plates were incubated in the dark at room temperature for 30 min (melatonin assay) or 25 min (cortisol assay). Within 10 min of adding Stop Solution, the optical density was read in Infinite 200 PRO (Tecan) at 450 nm. To obtain the best results, a secondary filter correction at 625 nm (melatonin assay) or 491 (cortisol assay) was performed. The concentration of melatonin and cortisol in the saliva samples was determined using a four-parameter non-linear regression curve fit. Melatonin levels were analyzed as baseline as well as second and fourth experimental sessions. Examples of melatonin profiles are presented in Supplementary Figure S2. The evening level of melatonin was calculated as an average of all five samples. The dim-light melatonin onset (DLMO) was calculated using the hockey-stick method (Danilenko et al., 2014). The cortisol awakening response (CAR; Stalder et al., 2016) was calculated by subtracting the concentration of salivary cortisol at waking from the average concentration at 30 and 60 min postwaking.

### Experimental Tasks

Participants performed a psychomotor vigilance task (PVT), a widely used test of sustained attention (Dinges and Powell, 1985; Basner and Dinges, 2011). The task required pressing a response button (with index finger) as soon as the stimulus appears, which stops the stimulus counter and displays the reaction time (RT) in milliseconds for a 1 s period. It was emphasized to the participants not to press the button in the absence of stimuli, which yielded a false start warning on the screen. If a reaction was slower than 1 s, the warning “too slow” appeared. The intertrial interval varied randomly from 2 to 10 s, and the task duration was 5 min, comprising ∼42 stimuli. The first three stimuli were discarded from the analysis in each PVT trial.

In the analysis, errors of commission were defined as responses without a stimulus or those with RT < 100 ms, whereas errors of omission as lack of response on stimulus or responses with RT ≥ 500 ms. The number of errors was calculated as a percentage of all stimuli in the task. For correct responses, mean RT, mean RT for 10% of the fastest responses, and mean RT for 10% of the slowest responses were calculated as the most frequently reported PVT outcome metrics (Basner and Dinges, 2011). Additionally, indicators of state instability, i.e., standard deviation of RT and the time-on-task effect, were tested and compared between sessions. To evaluate if there was a difference in the time-on-task effect across experimental sessions, the slope of linear regression line across RT for each minute of the task was calculated.

**FIGURE 3:**
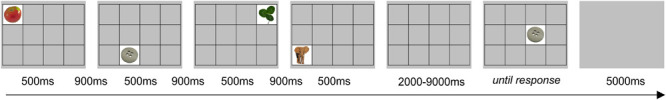
Example of trial for the sequential picture location task (SPLT). The item shown would require a response “no.”

To study visuospatial working memory, we used a sequential picture location task (SPLT; Figure 3). In the task, four pictures were sequentially presented in random square of the 4 × 3 grid for 500 ms with 900 ms interval between them. Participants were asked to remember the location of pictures. After a delay lasting from 2 to 9 s, a memory probe was presented at the screen until the response was given. The instruction was to respond “yes” (index finger) if the memory probe was in the same location as during the preceding sequence or “no” (middle finger) if the location was different. A 5 s blank screen was presented between trials. There were 56 trials that resulted in ∼15 min of task duration (actual task duration was dependent on participants’ response time). Only accuracy (in%) was measured since there was no time restriction on response. The pictures for the task were taken from the Bank of Standardized Stimuli (BOSS) database (Brodeur et al., 2010). Participants did not perform the task during the follow-up session. Nine of 37 participants (4 from BLB and 5 form CTRL group) performed shorter version of the task, i.e., there were 31 trials and 3 pictures to remember in each trial (those data were included in the analysis).

Both tasks were performed on a computer with 19-in LCD screen. For training and baseline session for the BLB group as well as for all experiment for the CTRL group, the blue-blocking filter was used on the screen to ensure similar visual conditions (i.e., color perception) for all participants (see filter properties in Figure 1). Participants responded with arrow keys on the keyboard.

### Statistical Analysis

The outcomes of the measures used in the experiment were tested if they show a changing pattern through consecutive sessions and if this pattern differs between groups. Thus, the interaction of *session* (five levels accounting for one baseline and four sessions of wearing BLB/regular contact lenses – a repeated measure within-subject factor) and *group* (two levels: BLP and CTRL group – between-subject factor) was checked in the two-factor mixed design (ANOVA) test. The Tukey honestly significant difference (HSD) test was used for *post hoc* comparisons. Tests were performed using Statistica software (version 13; StatSoft, Inc.). This way, the PVT outcomes, as well as actigraphy and questionnaire data and time spent outside during the day, were analyzed (note: for PSQI analysis, there were two levels for session factor, i.e., baseline and last experimental session).

The comparison of the PVT outcomes and ESS questionnaire in the follow-up session with baseline and the last week of wearing the BLB contact lenses was performed on data from the BLB group with repeated-measures ANOVA test. The goal of this analysis was to check whether any changes in behavior diminished ∼1 week after taking off the blue light blocking contact lenses. To test the time-on-task effect in the PVT task, additional factor (*minutes*) was introduced to ANOVA test. For the SPLT task, an additional between-subject factor was added to the ANOVA analysis in order to take into account the two versions of the task (note: none of the effects including *task version* factor was significant).

In the case of melatonin and cortisol data, the analysis was performed only on the BLB group. The repeated-measures ANOVA was used to test differences between sessions (baseline as well as second and fourth experimental sessions).

## Results

The comparison between groups was made in terms of age, gender, body mass index (BMI), sleep quality, level of daytime sleepiness, and chronotype for the group of 38 participants that completed the study [average age, 24.2 years (SD 3.9); 22 female; average BMI, 22.7 (SD 3.0)]. Data are summarized in Table 1. No difference was found between groups. The daily exposure to sunlight was estimated for each participant and each session taking into consideration self-reported average time spent outside in the week preceding the session as well as sunrise and sunset times in this week. There was no significant effect between groups and sessions [*F*_(__4_, _144__)_ = 1.96, *p* = 0.10; see Supplementary Figure S2]. In addition, there was no difference in refractive error between groups [*F*_(__1_, _72__)_ = 0.20, *p* = 0.66].

**TABLE 1:** Demographic and questionnaire data.

	**BLB group (*n*** = **19)**	**CTRL group (*n*** = **19)**	**Difference**
Females (nb)	11	11	*p* = 1.00
Age (years)	23.58 ± 2.76	24.89 ± 4.85	*p* = 0.31
PSQI	3.00 ± 1.16	3.00 ± 1.33	*p* = 1.00
ESS	5.90 ± 2.73	6.74 ± 2.05	*p* = 0.29
ChQ-ME	20.79 ± 5.63	20.37 ± 5.82	*p* = 0.82

**Data are reported as mean ± SD (except the number of female participants); statistical difference measured with t-test. PSQI, Pittsburgh Sleep Quality Index; ESS, Epworth Sleepiness Scale score; ChQ-ME, morningness–eveningness scale of Chronotype Questionnaire.**

Since the timing of the PVT and SPLT task differed between groups, we have checked the results from the baseline session. No significant differences were observed between groups neither for PVT [mean RT: *t*_(__36__)_ = 0.17, *p* = 0.86; mean RT for 10% of the fastest responses: *t*_(__36__)_ = 0.36, *p* = 0.72; mean RT for 10% of the slowest responses: *t*_(__36__)_ = 0.25, *p* = 0.80; number of omission errors: *t*_(__36__)_ = 1.13, *p* = 0.27] nor for SPLT task [*F*_(__1_, _33__)_ = 0.18, *p* = 0.67].

**FIGURE 4:**
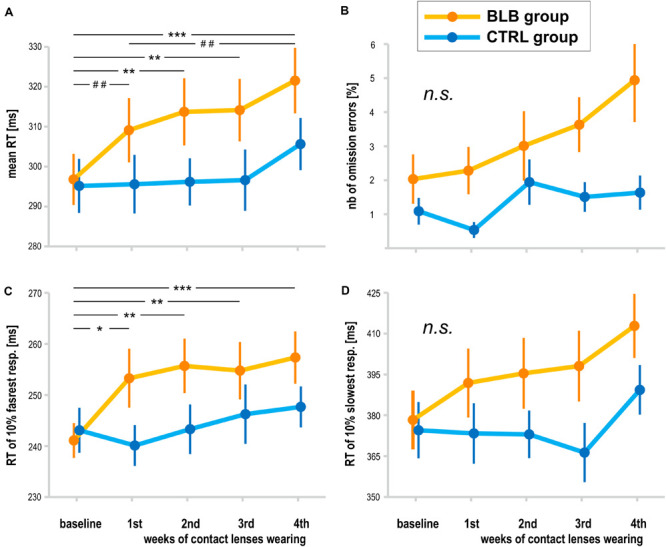
Psychomotor vigilance task (PVT) outcomes for the blue light blocked (BLB) and control (CTRL) groups for the baseline and 4 weeks of experimental condition: **(A)** mean reaction time, **(B)** number of omission errors, **(C)** reaction time for 10% of the fastest responses, and **(D)** reaction time for 10% of the slowest responses. ***p < 0.001; **p < 0.01; *p < 0.05; ^##^*p* < 0.1; bars indicate standard error.

The analysis of PVT data was performed on data obtained from 38 participants (19 from the BLB and 19 from the CTRL group). The outcome metrics are presented on graphs in Figure 4. Significant interaction effect of *session* and *group* factors was observed for mean RT [*F*_(__4_, _144__)_ = 2.59, *p* < 0.05, partial η^2^ = 0.07] and mean RT for 10% of the fastest responses [*F*_(__4_, _144__)_ = 2.80, *p* < 0.05, partial η^2^ = 0.07]. The Tukey HSD *post hoc* comparisons yielded a significant increase in these measures in consecutive sessions only for the BLB group; detailed test results are presented on the graphs (Figure 4). Other measures did not reach the significance level: mean RT for 10% of the slowest responses [*F*_(__4_, _144__)_ = 1.54, *p* = 0.19], number of omission errors [*F*_(__4_, _144__)_ = 1.37, *p* = 0.25], and number of commission errors [*F*_(__4_, _144__)_ = 1.51, *p* = 0.20].

The time-on-task effect, i.e., increasing RT in the course of the task, was present [*F*_(__4_, _144__)_ = 9.94, *p* < 0.001, partial η^2^ = 0.22]; however, interaction with *group* and *session* was not significant [*F*_(__16_, _576__)_ = 1.21, *p* = 0.25, partial η^2^ = 0.03]. The comparison of slopes did not reveal significant interaction between sessions and groups [*F*_(__4_, _144__)_ = 0.64, *p* = 0.63], neither the standard deviation of RT [*F*_(__4_, _144__)_ = 0.55, *p* = 0.70].

**FIGURE 5:**
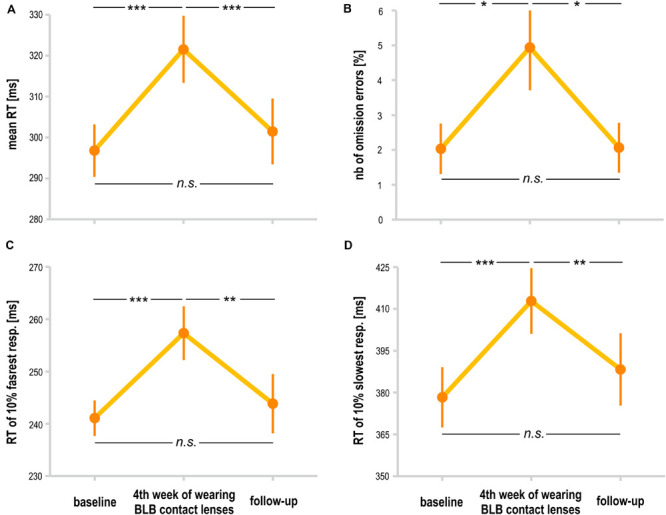
Comparison of psychomotor vigilance task (PVT) outcomes for the blue light blocked (BLB) group between baseline, fourth week of blue light reduction, and the follow-up session: **(A)** mean reaction time, **(B)** number of omission errors, **(C)** reaction time for 10% of the fastest responses, and **(D)** reaction time for 10% of the slowest responses. ***p < 0.001; **p < 0.01; *p < 0.05; bars indicate standard errors.

For the PVT task, we compared the follow-up session with the baseline and last week of wearing the BLB contact lenses for the BLB group (Figure 5). The repeated-measure ANOVA analysis revealed significant differences between those three sessions in mean RT [*F*_(__2_, _36__)_ = 21.79, *p* < 0.001, partial η^2^ = 0.55], mean RT for 10% of the fastest responses [*F*_(__2_, _36__)_ = 12.02, *p* < 0.001, partial η^2^ = 0.40], mean RT for 10% of the slowest responses [*F*_(__2_, _36__)_ = 13.58, *p* < 0.001, partial η^2^ = 0.43] and number of omission errors [*F*_(__2_, _36__)_ = 5.15, *p* < 0.05, partial η^2^ = 0.22]. The *post hoc* tests for all the above-mentioned analyses revealed significant difference between the baseline session and fourth week of wearing BLB contact lenses and between this session and the follow-up. There was no difference between the baseline and follow-up sessions. The number of commission errors did not show significant differences between sessions [*F*_(__2_, _36__)_ = 0.13, *p* = 0.88].

**FIGURE 6:**
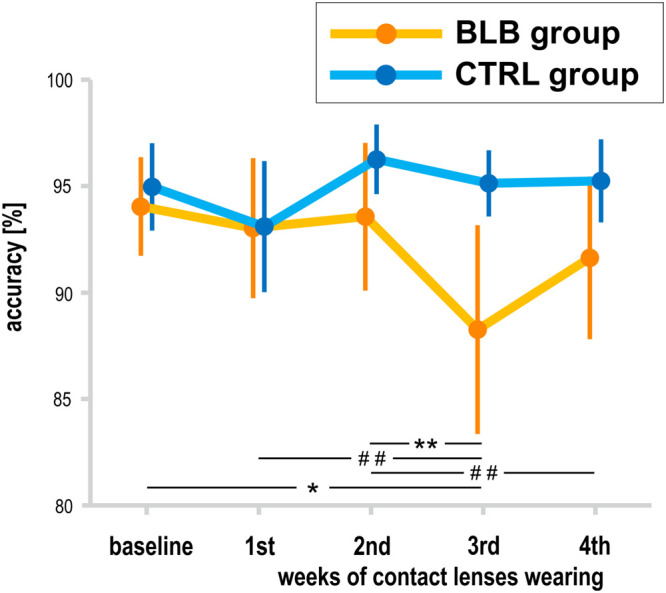
Accuracy in sequential picture location task (SPLT) for the blue light blocked (BLB) and control (CTRL) groups for the baseline and 4 weeks of experimental condition. **p < 0.01; *p < 0.05; ^##^*p* < 0.1; bars indicate standard errors.

The analysis of SPLT data was performed on data obtained from 37 participants (19 from the BLB and 18 from CTRL group); one participant from the CTRL group was excluded from the analysis due to low performance (<70% accuracy in all sessions). There was a significant interaction between *group* and *session* for the accuracy in the SPLT task [*F*_(__4_, _132__)_ = 3.77, *p* < 0.01, partial η^2^ = 0.10; Figure 6]. The *post hoc* test revealed significant decrease in accuracy only for the BLB group (see Figure 6).

Sleep parameters assessed with actigraphy are presented in Table 2. Actigraphy data from six participants (one from BLB and five from CTRL group) were not recorded for the whole experiment due to technical problems. There were no significant interaction for *group* and *session* factors for those measures [sleep onset: *F*_(__4_, _120__)_ = 0.68, *p* = 0.61; sleep offset: *F*_(__4_, _120__)_ = 0.40, *p* = 0.81; sleep latency: *F*_(__4_, _120__)_ = 0.24, *p* = 0.92; actual sleep time: *F*_(__4_, _120__)_ = 0.72, *p* = 0.58; intradaily stability: *F*_(__4_, _68__)_ = 0.88, *p* = 0.48; interdaily variability: *F*_(__4_, _68__)_ = 0.99, *p* = 0.42; relative amplitude: *F*_(__4_, _68__)_ = 1.76, *p* = 0.15].

**TABLE 2:** Actigraphy-derived sleep parameters in baseline and four weeks of experimental condition for the blue light blocked (BLB) and control (CTRL) group.

**Actigraphy measure**	**Group**	**Baseline**	**Weeks of contact lenses wearing**			
			**1st**	**2nd**	**3rd**	**4th**
Sleep onset	***BLB***	00:58 ± 1h27min	01:04 ± 1h53min	01:11 ± 2h14min	01:09 ± 2h10min	01:02 ± 1h56min
	***CTRL***	00:44 ± 50min	00:21 ± 43min	00:33 ± 1h11min	00:42 ± 1h9min	00:45 ± 51min
Sleep offset	***BLB***	08:15 ± 1h34min	08:24 ± 1h57min	08:43 ± 2h18min	08:45 ± 2h11min	08:28 ± 2h2min
	***CTRL***	07:53 ± 45min	08:01 ± 57min	08:00 ± 1h39min	08:10 ± 1h34min	08:02 ± 58min
Sleep onset latency	***BLB***	14min±13min	14min±12min	15min±13min	14min±12min	11min±10min
	***CTRL***	12min±9min	10min±8min	12min±8min	11min±7min	10min±7min
Actual sleep time	***BLB***	6h38min±39min	6h42min±52min	6h52min±1h5min	6h55min±38min	6h38min±47min
	***CTRL***	6h46min±1h8min	7h12min±1h8min	6h59min±1h16min	6h58min±1h11min	6h48min±56min
Intradaily stability*	***BLB***	0.79 ± 0.33	0.79 ± 0.24	0.83 ± 0.28	0.79 ± 0.25	0.90 ± 0.17
	***CTRL***	0.37 ± 0.13	0.45 ± 0.07	0.42 ± 0.12	0.47 ± 0.12	0.43 ± 0.11
Interdaily variability*	***BLB***	0.60 ± 0.32	0.57 ± 0.28	0.54 ± 0.26	0.54 ± 0.21	0.45 ± 0.16
	***CTRL***	0.86 ± 0.24	0.86 ± 0.30	0.90 ± 0.24	0.82 ± 0.22	0.89 ± 0.21
Relative amplitude*	***BLB***	0.87 ± 0.08	0.84 ± 0.14	0.90 ± 0.07	0.89 ± 0.05	0.83 ± 0.10
	***CTRL***	0.75 ± 0.21	0.90 ± 0.05	0.88 ± 0.16	0.89 ± 0.08	0.83 ± 0.11

*Measures derived from non-parametric circadian analysis on MotionWatch8 data from 19 participants.

**FIGURE 7:**
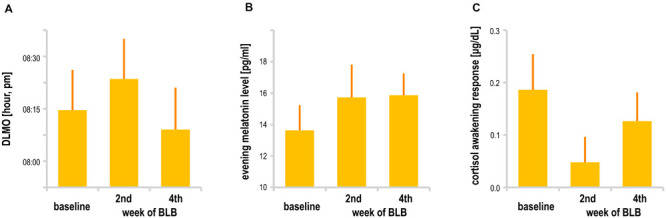
**(A)** Dim light melatonin onset, **(B)** mean evening melatonin concentration, and **(C)** cortisol awakening response for the blue light blocked (BLB) group in the baseline, second, and fourth week of blue light reduction.

Melatonin data did not show significant effect of session [mean evening melatonin level, *F*_(__2_, _36__)_ = 1.99, *p* = 0.15; DLMO, *F*_(__2_, _26__)_ = 1.68, *p* = 0.21]. In addition, cortisol data in terms of CAR did not change between sessions [*F*_(__2_, _34__)_ = 1.72, *p* = 0.20]. However, one may observe a slight increase in mean evening melatonin levels after 2 weeks of BL blockade as well as weaker morning cortisol response after this time; those trends do not continue in the second half of the experiment. The results are presented in Figure 7. The analysis was performed only for the BLB group. For four subjects, there was an insufficient number of data points for DLMO calculation due to missing data or melatonin levels below the sensitivity threshold. For one subject, onset could not be calculated due to stable melatonin level; thus, DLMO results are reported for 14 participants. The CAR was calculated for 18 participants (missing samples for 1 subject).

**FIGURE 8:**
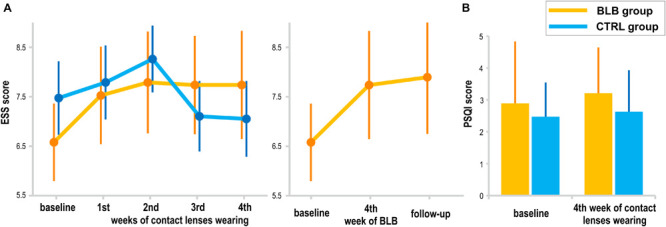
Subjective measures of sleepiness and sleep quality: **(A)** Epworth Sleepiness Scale (EES) score for the blue light blocked (BLB) and control (CTRL) groups in consecutive experimental sessions as well as comparison to follow-up session for BLB group, **(B)** Pittsburgh Sleep Quality Index (PSQI) score at the baseline session and after 4 weeks of experimental condition for both groups. Bars indicate standard errors.

The subjective daytime sleepiness (ESS) and sleep quality (PSQI) results are presented in Figure 8. The interaction effect of *session* and *group* factors in the ANOVA test for both scores was not significant [ESS, *F*_(__4_, _144__)_ = 1.72, *p* = 0.15; PSQI, *F*_(__1_, _36__)_ = 0.12, *p* = 0.73]. When considering the follow-up session, the ESS score did not show significant effect [*F*_(__2_, _36__)_ = 2.24, *p* = 0.12]. The questionnaire results are reported for 38 subjects (19 from the BLB and 19 from the CTRL group).

## Discussion

In this study, we investigated the effects of prolonged blocking of blue light on sustained attention and visuospatial working memory as well as on circadian rhythmicity. We used the PVT, a simple reaction time task that indicates the ability to achieve and maintain a certain level of cognitive performance (Souman et al., 2018), as well as the SPLT task requiring memory both for object and spatial locations. Actigraphy was applied to control for timing and duration of sleep, PSQI for assessing its quality, and ESS to control for daytime sleepiness levels. The melatonin and cortisol levels were also assessed. We compared the data collected from two groups of participants wearing either contact lenses with filter blocking ∼90% of blue light or normal contact lenses during four consecutive weeks. Results showed a significant change of pattern in the majority of the PVT outcome metrics in the BLB group. Particularly, the longer blocking the blue light lasted, the slower the responses as indicated by mean RT and mean of the 10% fastest responses. The effects on other PVT measures did not reach significance level, although they also presented an increasing pattern in the course of weeks with reduced blue light. In the CTRL group, all the metrics were stable through all sessions. There was no effect of changed light condition on RT variability measured by standard deviation as well as on time-on-task effect. The comparison between the baseline session and the last experimental session in the BLB group revealed a significant increase in all of the PVT outcome and a significant decrease in those measures at the follow-up session. In the case of the SPLT task, a significant decrease in accuracy was shown only for the BLB group. Sleep timing and length, subjective sleep quality, and daytime sleepiness did not change during the experimental intervention. No effect was observed for melatonin levels, dim light melatonin onset, and cortisol levels. These negative results may suggest that people adapt quickly to lowered levels of BL; that is, even low exposure (i.e., 10% of the regular level) may regulate circadian rhythmicity of sleep and hormones’ secretion. The result is in accordance with the conclusions drawn by Giménez et al. (2014) who showed that melatonin suppression was actually reduced immediately after limiting blue light transmittance by using orange-tinted contact lenses but not after 2 weeks of wearing them. Contrary, it was shown that prolonged exposure to significantly dimmed light with dark goggles for a week (Hébert et al., 2002) or in laboratory conditions for 3 days (Smith et al., 2004) increases the sensitivity of melatonin secretion system (i.e., increase in melatonin suppression). However, in these experiments, the whole spectrum of light was reduced for a shorter period comparing to our study as well as Giménez et al. (2014). The adaptive mechanisms were also suggested by Najjar et al. (2014). Here, decreased light transmittance in aged lenses and thus changed light history was associated with a shift of non-visual sensitivity to longer wavelengths. An alternative interpretation of our findings involves the impact of social zeitgebers, so strong in everyday life that sleep–wake timing stays constant, and this regulates overall circadian rhythmicity.

As hypothesized, our study showed that blocking blue light slows down reactions in sustained attention task and causes deterioration of performance in the visuospatial memory task. This effect gradually strengthens in consecutive weeks. Interestingly, our results revealed that in case of sustained attention, this effect is reversible, as the reaction times decrease to the baseline level after returning to “normal” light conditions (i.e., with BL in the spectrum).

The effect of blue light on cognitive processes may be considered in terms of complex interactions between circadian, sleep, and arousal factors. Particularly, blue light, through the NIF system, can either act directly on the neuronal system and thus on alertness and behavior, or indirect effects may occur due to disrupted entrainment of the circadian system and/or sleep (Fisk et al., 2018). Therefore, the effects of the changed blue light conditions can be interpreted in terms of different mechanisms.

Based on previous studies on sleep–wake regulation, it can be stated that blocking blue light causes similar outcomes to those of sleep deprivation (Lim and Dinges, 2008). Thus, performance decrement reported in our study might be linked to disruption of sleep or sleep–wake rhythm. However, in contrast to research on sleep deprivation, our measurements imply that participants had no sleep problems in conditions of blue light filtering. Neither sleep duration nor sleep quality was affected. Taking into account the direct impact of light on melatonin secretion and circadian regulation (Touitou et al., 2017), one could expect, in conditions of the blue light blockade, the following changes in participants’ sleep–wake pattern: earlier bedtime, shorter sleep latency, and longer sleep. This could lead to improved well-being due to better sleep. However, it may be also the case that lack of triggering effect of blue light would be the key factor of daytime drowsiness, hence lack of energy and decreased well-being. In our study, 8 out of 18 participants (44%) showed earlier sleep onset after 4 weeks of experiment (so did 5 out of 14 controls, i.e., 36%), 35% of experiment participants (vs. 43% of controls) exhibited shortened sleep latency, and 39% of participants (vs. 36% of controls) had longer sleep. Those results do not support the above assumptions on the direct link between blue light and sleep–wake rhythm. In general, the prolonged blue light reduction did not result in significant changes in sleep pattern, although we are aware of the fact that the “pristine” internal sleep timing is influenced by external social factors that are not controlled in case of an experiment conducted in “natural” conditions. The results obtained by Giménez et al. (2014), who reduced BL exposure for 2 weeks, did not show differences in the timing of sleep, its efficiency, and subjective quality; likewise, no effect was found on dim light melatonin onset or on the amplitude of melatonin rhythms. Thus, the unchanged sleep-related indicators in our study may be interpreted as speaking for lack of BLB effect on sleep–wake rhythm and sleep *per se* and in consequence on performance through this indirect pathway. The unchanged DLMO as well as evening melatonin and morning cortisol levels corroborates this assumption.

Therefore, the results of our study could be interpreted in terms of the direct effect of light on alertness. The series of neuroimaging experiments by Vandewalle and colleagues demonstrated that blue light induces modulations of brain activity while participants are engaged in non-visual cognitive tasks (Vandewalle et al., 2007a, b, 2009). Those activations regarded alertness-related subcortical structures such as the brainstem, hypothalamus, dorsal and posterior parts of the thalamus, hippocampus, and amygdala. The modulations were detected also in the cortex, in areas involved in the bottom–up reorientation of attention and in the regions linked with top–down regulation of attention. At the behavioral level, blue-enriched light was shown to enhance subjective alertness and led to significantly faster reaction times in tasks associated with sustained attention and working memory (Chellappa et al., 2011b; Alkozei et al., 2016). Our experimental condition was constructed in the opposite way, as we reduced blue light exposure and so we observed increasing reaction times and decreasing accuracy. Hence, the reduced amount of blue light reaching the retina may be associated with insufficient stimulation of the alerting and/or orienting system in the brain that, in consequence, may have an impact on cognitive processes.

Prolonged filtering of the blue light is a part of the aging process of the eye. As known, aging is often associated with sleep and circadian disturbances (Duffy and Czeisler, 2002) as well as cognitive decline (Cabeza et al., 2016). Our results suggest that a reduced amount of blue light reaching the retina might be one of the factors influencing the deterioration of cognitive functioning. A finding by Schmoll et al. (2011) supports this explanation. They examined patients after cataract surgery and revealed that improved blue light transmission had a beneficial effect on cognitive function (responses became both quicker and more consistent following surgery). Moreover, the detrimental effect of blocking blue light can be reversed as demonstrated in our study (returning to the baseline level of task performance after returning to normal light conditions) or attenuated as shown in studies on elderly population (a long-term, whole-day bright light exposure in a large cohort of care facilities residents (Riemersma-van der Lek et al., 2008).

### Limitations

A methodological limitation of our study is the fact that the participants from each group performed tasks at different times (i.e., control group ∼2 h earlier than experimental group). This time difference could potentially introduce a bias in the results. However, as stated in sections “Materials and Methods” and “Results,” we have compared the results from the baseline session between groups and found no significant differences. It is important to note that the time of task performance was the same in every session for each group; thus, bias regarding circadian and/or homeostatic factors was eliminated in the comparison between subsequent experimental sessions. Nevertheless, the timing of testing could alter the effect of prior light history on performance in both tasks.

Our experiment was conducted in the so-called “natural settings.” The participants did not stay in the lab, and we did not impose on them a specific sleep–wake schedule. Therefore, we had no control over all potentially disruptive factors that could affect the sleep–wake cycle and, consequently, the psychophysical state. However, the activity was controlled with the use of actigraphs and sleep parameters calculated from these recordings differed neither between sessions nor between groups.

Because of organizational constraints, we were not able to set individual timetables for every participant, taking into account their habitual wake- and bedtimes. We decided to collect the saliva samples at most common hours (from 7 to 11 P.M.), risking that, in some cases, it might not cover the time of melatonin rise and those data would be unusable. Indeed, we noticed a wide range of sleep times in our participants and the problem with determining DLMO happened with one subject.

## Conclusion

In summary, we found that prolonged and substantial reduction of blue light causes a worsening of performance in sustained attention and visuospatial memory tasks. At the same time, no effects on sleep parameters were observed. The lack of significant changes in sleep pattern and melatonin indicators during a 4-week experiment may be an indirect proof of the stability of sleep–wake rhythm. Thus, the observed deterioration of cognitive functioning was not related to indirect effects through disruption of the circadian system, but rather, it may be directly attributed to a lack of boosting effect of blue light.

## Data Availability Statement

The raw data supporting the conclusions of this article will be made available by the authors, without undue reservation.

## Ethics Statement

The studies involving human participants were reviewed and approved by the Bioethics Commission at the Polish Military Institute of Aviation Medicine. The patients/participants provided their written informed consent to participate in this study.

## Author Contributions

AD, HO, EB, MF, PC, MR, and TM: conception and design of the work. AD, HO, and MP: acquisition and analysis. AD, HO, EB, MF, and TM: interpretation of the results. AD: drafting the work. AD, HO, EB, MF, MP, PC, MR, and TM: revising the manuscript critically and final approval of the version to be published. All authors contributed to the article and approved the submitted version.

## Conflict of Interest

The authors declare that the research was conducted in the absence of any commercial or financial relationships that could be construed as a potential conflict of interest.
