# Supplementary material for: Long-Term Reduction of Short-Wavelength Light Affects Sustained Attention and Visuospatial Working Memory With No Evidence for a Change in Circadian Rhythmicity
Source: Front Neurosci. 2020 Jul 3;14:654. doi: 10.3389/fnins.2020.00654 (PMC7348134; doi:10.3389/fnins.2020.00654)
Supplement: Supplementary file 1 [file Table_1.DOCX]

Supplementary Material

# Supplementary Figures


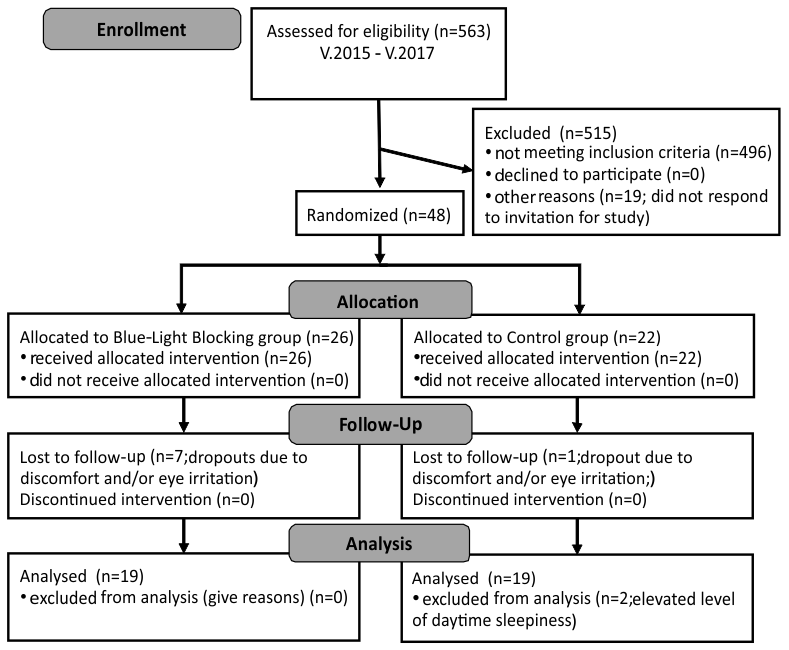


**Supplementary Figure 1.** Flow chart of participant recruitment.


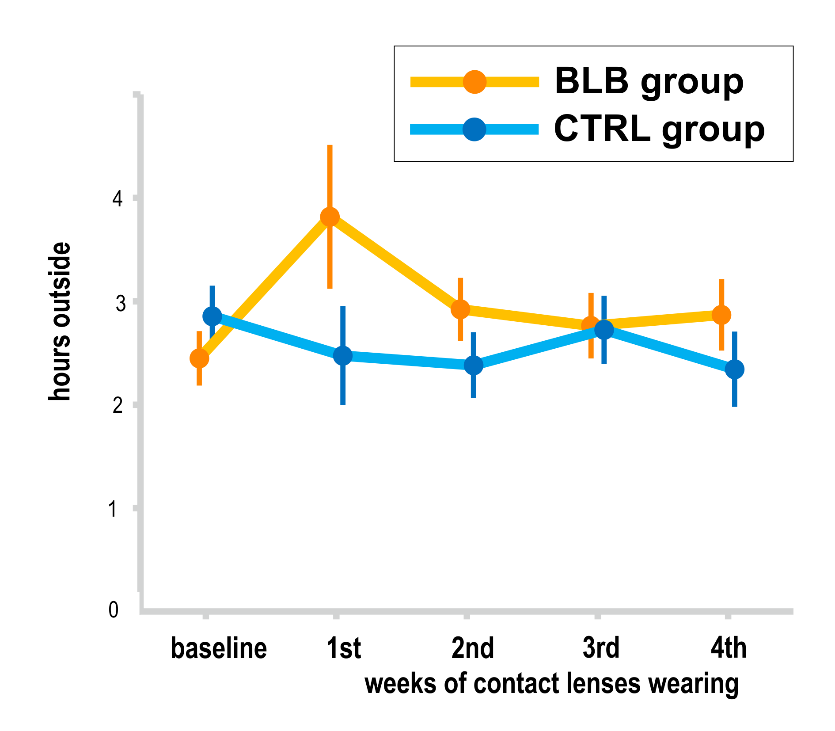


**Supplementary Figure 2.** Estimated daily exposure to sunlight.

**
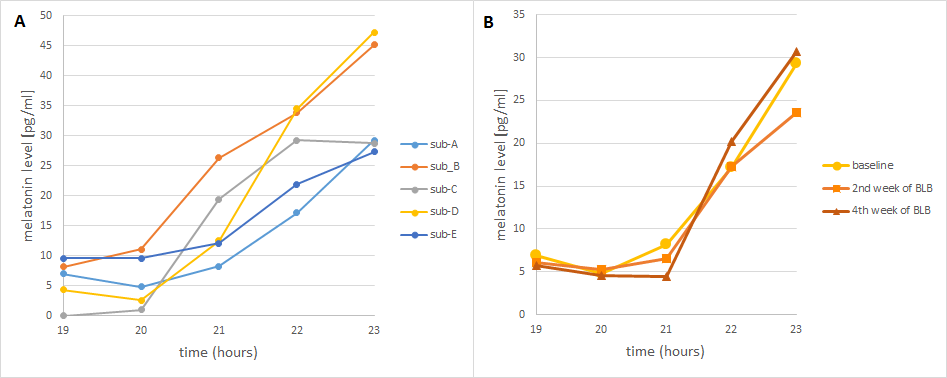
**

**Supplementary Figure 3.** Examples of melatonin profiles at baseline (A) and across the study for one subject (B).
